# Lexical frequency effects on articulation: a comparison of picture naming and reading aloud

**DOI:** 10.3389/fpsyg.2015.01571

**Published:** 2015-10-15

**Authors:** Petroula Mousikou, Kathleen Rastle

**Affiliations:** Department of Psychology, Royal Holloway, University of LondonEgham, UK

**Keywords:** speech production, reading aloud, picture naming, articulation, acoustics, reaction times

## Abstract

The present study investigated whether lexical frequency, a variable that is known to affect the time taken to utter a verbal response, may also influence articulation. Pairs of words that differed in terms of their relative frequency, but were matched on their onset, vowel, and number of phonemes (e.g., *map* vs. *mat*, where the former is more frequent than the latter) were used in a picture naming and a reading aloud task. Low-frequency items yielded slower response latencies than high-frequency items in both tasks, with the frequency effect being significantly larger in picture naming compared to reading aloud. Also, initial-phoneme durations were longer for low-frequency items than for high-frequency items. The frequency effect on initial-phoneme durations was slightly more prominent in picture naming than in reading aloud, yet its size was very small, thus preventing us from concluding that lexical frequency exerts an influence on articulation. Additionally, initial-phoneme and whole-word durations were significantly longer in reading aloud compared to picture naming. We discuss our findings in the context of current theories of reading aloud and speech production, and the approaches they adopt in relation to the nature of information flow (staged vs. cascaded) between cognitive and articulatory levels of processing.

## Introduction

Speech production involves the combination of cognitive and articulatory processes. However, these processes have been traditionally investigated in separate domains of research, yielding a division between models of speech production that focus on psycholinguistic (e.g., Dell, 1986; Levelt et al., 1999) vs. motor control (Guenther et al., 2006) aspects of this process. This division is likely due to the widely held assumption that the transition from cognitive to articulatory levels of processing occurs in a *staged* manner, so that articulatory processes can only be initiated after cognitive processing is complete (Levelt et al., 1999). On this assumption, the articulation of an utterance should be unaffected by higher-level cognitive processes that are involved in selecting an abstract phonological code for speech production. However, several studies to date have shown that articulation is affected systematically by such higher-level processes (see Bell et al., 2009, and Gahl et al., 2012, for comprehensive reviews). The results of these studies suggest that articulation can be initiated before higher-level processes involved in the selection of a phonological code are finished. This finding offers support for the view that information from cognitive to articulatory levels of processing flows in a *cascaded* manner.

More specifically, these studies used different approaches to investigating whether high-level cognitive processes cascade down to articulation. Such approaches involved examining the nature of *speech errors* and showing that erroneous productions contain articulatory features of the non-produced target sound. This finding was thought to indicate that partial information from the target sound can cascade into articulation. Other approaches involved examining how certain *lexical variables* such as word frequency and phonological neighborhood density may influence articulatory detail, or how *syntactic predictability* and *semantic congruency* may affect articulation. Each of the four approaches is further elaborated below.

### Speech errors

Speech errors induced in the laboratory often reflect the simultaneous production of competing sounds. Goldrick and Blumstein (2006) designed a tongue twister task in which participants had to repeat a sequence of syllables (e.g., *keff geff geff keff*) at a rate faster than normal speech. They found that when participants erroneously produced /g/, there were phonetic traces of the target sound /k/. In these instances, /g/ had a longer Voice Onset Time (VOT) (i.e., it was more /k/-like) than correctly produced /g/ sounds. This finding shows that partial activation of both the target sound and the competing sound is reflected in the articulation of the spoken output. Hence, unselected phonemic representations can influence articulatory detail.

Similarly, in a study that used Electromagnetic Articulography (EMA) participants were asked to repeat as quickly as possible the phrase *top cop* (Goldstein et al., 2007). The results from this study showed that the articulatory gestures associated with the sounds /t/ and /k/ (i.e., raising of the tongue tip and the tongue body) were produced simultaneously. Similarly, Pouplier (2007) asked participants to read silently word pairs with a specific consonant order in their onsets (e.g., ***g****ap* ***d****upe*, ***g****ob* ***d****ub*, ***g****um* ***d****am*) before they were unexpectedly asked to pronounce a word pair with opposite consonant order (***d****ome* ***g****imp*). Participants' productions revealed that the tongue tip was high during the initial /d/ in *dome*, but the tongue dorsum also displayed unexpected raising, which is characteristic of the articulatory gesture associated with /g/. Taken together, these results indicate that the partial activation of competing sounds cascades down to articulation.

Last, using a tongue-twister paradigm, McMillan and Corley (2010) asked participants to read groups of four ABBA syllables, where A and B differed in the onset by a voice feature (e.g., *kef gef gef kef*); a place feature (e.g., *kef tef tef kef*); both voice and place features (e.g., *kef def def kef*); or were identical (e.g., *kef kef kef kef*). Participants' responses were not categorized as “correct” or “wrong”; instead, the variability in the articulation of participants' *kef* productions during error invoking conditions (e.g., *kef gef gef kef, kef tef tef kef, kef def def kef*) were investigated relative to the baseline condition (e.g., *kef kef kef kef*). The results from this study showed significantly more articulatory variability in the VOT productions when the onsets of the A and B syllables differed in voice only (e.g., *kef gef gef kef*). There was also more articulatory variability in lingual contact with the palate, measured with Electropalatography (EPG), when the onsets of the A and B syllables differed only in place of articulation (e.g., *kef tef tef kef*). Moreover, articulatory variability of both VOT and location of palate contact was significantly smaller when the onsets of the A and B syllables differed in both place and voice (e.g., *kef def def kef*). The results from this study provide further evidence in favor of the idea that properties of phonologically-similar competing utterances cascade into articulation.

### Lexical effects

High-frequency (HF) words are typically produced with shorter durations, reduced vowels, deleted codas, more tapping and palatalization, and reduced pitch range, compared to low-frequency (LF) words (e.g., Zipf, 1929; Fidelholz, 1975; Hooper, 1976; Rhodes, 1992, 1996; Fosler-Lussier and Morgan, 1999; Kawamoto et al., 1999; Bybee, 2000; Munson and Solomon, 2004; Pluymaekers et al., 2005a; Aylett and Turk, 2006; Gahl, 2008). For example, Pluymaekers et al. (2005a) used data from a corpus of spontaneous speech in Dutch to examine the production of the same affixes appearing in different words that varied in frequency. They observed that suffixes belonging to HF words were more reduced than those belonging to LF words. Using data from the Switchboard corpus of American English telephone conversations, Gahl (2008) also reported that HF English homophones (e.g., time) were produced with shorter durations than their LF counterparts (e.g., thyme). Accordingly, in a reading aloud task, Munson and Solomon (2004) observed that vowels in LF words were produced with longer durations and closer to the periphery of the vowel space (hence, with more extreme articulation) than vowels in HF words. Initial-phoneme durations were also found to be longer for LF words in reading aloud (Kawamoto et al., 1999), which led the authors to conclude that the criterion to initiate pronunciation is based on the initial phoneme and not on the whole word. This finding challenges the assumption that articulation is initiated only after phonological encoding is complete (Levelt et al., 1999). Taken together, these results suggest that *lexical frequency*, a variable that has been traditionally known to affect high-level cognitive processes, also affects low-level articulatory processes.

Words from dense neighborhoods (i.e., words which are phonologically similar with several other words) are hyperarticulated in reading aloud, compared to words from sparse neighborhoods (Wright, 1997, 2004; Munson and Solomon, 2004; Munson, 2007; but see Gahl et al., 2012, who observed that words from dense neighborhoods were phonetically reduced in spontaneous speech). In particular, in these studies, vowels in words from high-density neighborhoods were produced closer to the periphery of the vowel space (hence, with extreme articulation), whereas vowels in words from sparse neighborhoods were produced closer to the center of the vowel space. Accordingly, Baese-Berk and Goldrick (2009) observed that words with minimal pair onset neighbors (e.g., *cod-god*) were produced with more extreme VOTs (hence, were more hyperarticulated) than words with no minimal pair onset neighbors (e.g., *cop-gop*, where *gop* is a non-word). Last, Scarborough (2004) found that vowels in LF words from high-density neighborhoods were more coarticulated than vowels in HF words from low-density neighborhoods. Although this finding seems to contradict previous findings, Scarborough (2004) took this result to indicate that speakers coarticulate the vowels more in words that are harder for listeners to recognize in order to facilitate lexical access (Luce and Pisoni, 1998). Taken together, these findings suggest that similarly to lexical frequency, *phonological neighborhood density* influences articulation.

### Syntactic predictability effects

Words that are predictable in a sentence are produced with shorter durations and more reduced vowels (e.g., Lieberman, 1963; Liu et al., 1997; Griffin and Bock, 1998; Krug, 1998; Bybee and Scheibman, 1999; Gregory et al., 1999; Jurafsky et al., 2001; Aylett and Turk, 2004; Pluymaekers et al., 2005b). Further, repeated words (e.g., words that have occurred in a previous sentence) are more predictable, thus they tend to be shortened (e.g., Fowler and Housum, 1987; Fowler, 1988; Hawkins and Warren, 1994; Bard et al., 2000). Finally, words in less probable syntactic constructions are produced with longer durations (e.g., Gahl and Garnsey, 2004; Gahl et al., 2006; Tily et al., 2009). Taken together, these findings suggest that *syntactic predictability* influences articulatory detail.

### Semantic congruency effects

Balota et al. (1989) observed that words that were cued by semantically congruent primes (e.g., *dog* preceded by *cat*) were produced with shorter durations compared to when these words were cued by semantically incongruent primes (e.g., *pen*). Using the Stroop paradigm, Kello et al. (2000) asked participants to name the color of rectangles with superimposed distractor words that were either semantically congruent, incongruent, or neutral (i.e., if the rectangle was colored in red the congruent condition consisted of the superimposed word *red*; the incongruent condition consisted of the superimposed word *blue*, and the neutral condition consisted of the superimposed letter string *iiiii*). The results from this study showed a Stroop interference effect, so that the incongruent condition yielded significantly slower color-naming latencies compared to the neutral condition. In addition, when participants had a deadline within which they had to respond, color naming durations were significantly longer in the incongruent condition relative to the neutral condition. These findings support the idea that *semantic congruency*, another variable that is thought to affect high-level cognitive processes, also influences articulation.

However, the empirical evidence in this research domain is not entirely consistent. Meyer (1990), for example, observed that single words were produced faster when they occurred in a phonologically similar context, yet their durations were unaffected by the context in which they occurred. Similarly, Schriefers and Teruel (1999) found that naming latencies of adjective-noun utterances (e.g., *red house*) were affected by distractor words that were phonologically related to the adjective, yet the durations of either the adjectives or the nouns were unaffected by the same experimental manipulation. Moreover, using three different speech production paradigms, a picture-word interference task with semantic and phonological relatedness between pictures and distractors, a picture-naming task in which pictures were blocked either by semantic category or by word-initial overlap, and a Stroop task, such as that used by Kello et al. (2000), Damian (2003) found no evidence for the idea that central cognitive processes influence articulation once a response has been initiated. As such, he argued that “articulation is not affected by prior processing stages—a finding that is easily accommodated by theoretical approaches that clearly separate articulation from preceding stages” (Damian, 2003, p. 429).

More recently, Riès et al. (2012, 2014) sought to determine the reason why naming pictures takes longer than reading aloud words. According to the literature in this domain, this is so because access to semantic information, which is required in picture naming but not necessarily in reading aloud, is time-consuming (Theios and Amrhein, 1989). In addition, it has been suggested that the stimulus-response association is equivocal in picture naming (i.e., some pictures may receive more than one name) but not in word reading aloud, thus yielding response uncertainty in the former task but not in the latter (Ferrand, 1999). These explanations imply that the response latency differences observed in the two tasks are due to differences in the processes that are involved in word-selection in the two tasks. However, verbal response latencies reflect not only the time that is required to select a word, but also the time to plan and initiate articulation. As such, the response latency differences observed in the two tasks could be due to a delay in planning and initiating articulation in picture naming compared to word reading aloud. If this hypothesis is true, strong evidence will be provided in favor of the idea that task-inherent cognitive processes (e.g., activation of semantic information, response uncertainty) cascade into articulation. Riès et al. (2012, 2014) tested this hypothesis using a reaction-time (RT) fractionation procedure in a reading aloud and a picture-naming task. RT was defined as the delay between stimulus presentation and the onset of the verbal response. Electromyographic (EMG) activity from several lip muscles was also recorded. The stimulus-response (SR) interval was divided into a premotor interval (from stimulus onset to EMG activity) and a motor interval (from EMG activity to verbal response). The results from the Riès et al. (2014) study showed that the difference between picture naming and reading aloud times was due to the premotor interval. This finding is consistent with Damian's (2003) results falsifying the theory that high-level cognitive processes affect articulatory processes.

In the present study, we re-examined this idea. In particular, we investigated whether lexical frequency affects initial-phoneme durations in picture naming and reading aloud. Lexical frequency is known to affect the time taken to select a phonological code for production. However, if it also influences durational aspects of the verbal response, we can conclude that cognitive processing is taking place after the verbal response is initiated. Such a finding will imply that information from cognitive to articulatory levels of processing flows in a cascaded manner. In contrast, if lexical frequency does not have an effect on durational aspects of the verbal response, we can conclude that processing at high cognitive levels is completed before the verbal response is initiated, and so the nature of information flow between cognitive and articulatory levels of processing must be staged. On the basis of previous results in the literature, we predicted that LF items would yield longer initial-phoneme durations than HF items.

In addition, we examined effects of lexical frequency on response times. Based on previous findings, we predicted that LF items would yield slower response times than HF items. Furthermore, we hypothesized that lexical frequency effects on verbal responses should be more prominent in picture naming than in reading aloud. This is because semantic activation of the target stimulus is required in picture naming; hence, its associated lexical frequency will have a robust effect on verbal responses. In contrast, reading aloud of a printed word can be performed, in principle, on the basis of sublexical information, and so lexical frequency effects on verbal responses are likely to be attenuated in this task. Accordingly, we predicted that both in the reaction time analyses and the analyses of initial-phoneme durations, the frequency effect would be bigger in size in picture naming than in reading aloud.

Last, we examined task effects on whole-word durations. Hennessey and Kirsner (1999) found that the same words were produced with longer durations in reading aloud compared to picture naming. They posited that reading aloud may be initiated on the basis of sublexical information (e.g., initial phoneme), and so processing of the rest of the word must be carried out during response execution, thus elongating response durations in this task compared to picture naming (see also Damian, 2003, and Kawamoto et al., 1998, for a similar account). Yet, this explanation is at odds with the idea that reading aloud begins when the computation of phonology is complete (Rastle et al., 2000). The present study further allows us to test these opposing views.

A common assumption in one of the most prominent psycholinguistic models of speech production (e.g., Levelt et al., 1999) is that the transition from cognitive to articulatory levels of processing during speech occurs in a staged (rather than a cascaded) manner, and so articulation can only be initiated after cognitive processing is complete. Similarly, the most prominent models of single word reading aloud (e.g., the DRC model of Coltheart et al., 2001; the CDP+ model of Perry et al., 2007; and the PDP model of Plaut et al., 1996) make the assumption that reading aloud cannot be initiated unless the orthography-to-phonology conversion of the printed letter string is complete. Thus, the results from the present study are critical for the evaluation of extant theories of speech production and reading aloud.

## Method

### Participants

Sixty undergraduate students from Royal Holloway, University of London, were paid £5 to participate in the study. Thirty of them participated in the picture naming task and the other 30 participated in the reading aloud task. Participants were monolingual native speakers of Southern British English and reported no visual, reading, or language difficulties.

### Materials

In order to make the picture naming and reading aloud tasks as comparable as possible the same items were used in both tasks. The selected items (*N* = 72) were between three and six letters long, had three or four phonemes, and had a CVC or CCVC structure. They were all regular words (i.e., with consistent spelling-to-sound mappings) that could be depicted as concrete objects.

The 72 items comprised 36 pairs of words that differed in their relative frequency, but were matched on number of phonemes and shared the same onset and vowel (e.g., *map* vs. *mat* and *brain* vs. *braid*, where *map* and *brain* are more frequent than *mat* and *braid*, respectively). Matching these pairs of words on their onset and vowel was important insofar as frequency effects on articulation were measured in terms of initial-phoneme durations, which are known to vary as a function of the identity of the following vowel or consonant (Klatt, 1975). Two lists were created using these word pairs, with one list containing items that were significantly higher in frequency than the items in the other list [*t*_(35)_ = 8.27, *p* < 0.001]^1^. Age of acquisition (AoA) is known to have a robust effect on picture naming latencies that is independent of the frequency effect (see Bates et al., 2001; Meschyan and Hernandez, 2002). For this reason, we ensured that the items in the HF list had significantly lower AoA than the items in the LF list [*t*_(35)_ = −4.42, *p* < 0.001]. AoA values were obtained from Kuperman et al. (2012). The two lists were additionally matched on orthographic neighborhood, which was measured in terms of total orthographic neighbors [*t*_(35)_ = −1.02, *p* > 0.05] and substitution orthographic neighbors [*t*_(35)_ = −1.57, *p* > 0.05]; and phonological neighborhood, which was also measured in terms of total phonological neighbors [*t*_(35)_ = 0.45, *p* > 0.05] and substitution phonological neighbors [*t*_(35)_ = −0.22, *p* > 0.05]. The orthographic and phonological neighborhood information was extracted from the CLEARPOND database (Marian et al., 2012). The means of each of the linguistic variables for the HF and LF items are presented in Table 1. The paired words are provided as Supplementary Material.

**Table 1:** **Characteristics of the items used in the picture naming and reading aloud tasks**.

	**High frequency**	**Low frequency**
SUBTLEX-UK log frequency (Zipf scale)	4.7	3.8
SUBTLEX-US frequency (per million)	60.6	10.0
Age of Acquisition	4.5	6.1
Number of letters	4.1	4.0
Number of phonemes	3.3	3.3
Orthographic neighbors (total)	14.5	15.6
Orthographic neighbors (substitution)	10.4	11.8
Phonological neighbors (total)	28.6	27.9
Phonological neighbors (substitution)	22.8	23.3

The 72 pictures consisted of black-and-white line drawings of common objects. Most pictures were selected from the IPNP (International Picture Naming Project) database (Szekely et al., 2004) and the remaining were obtained from different sources, yet they were all comparable in style^2^. The pictures varied slightly in width (226–400 pixels) and height (144–400 pixels) to avoid distorting the original shape of the depicted object; however, the longest side of each picture never exceeded 400 pixels and all pictures appeared in the center of the screen.

### Design

In the picture naming task, each participant underwent a training phase and a test phase. The training phase consisted of two parts. During the first part, participants were told that the aim of this first training phase was to become familiar with the names of a set of pictures that they would be asked to name later. On each trial, participants saw a picture appearing on the computer screen and heard its corresponding name via headphones. The names of the pictures had been recorded by a female native speaker of Southern British English. Participants studied each picture for as long as they needed, and controlled the time at which the next picture was presented with a button press. The 72 pictures were presented to each participant in a different random order. During the second part of the training phase, we assessed whether participants remembered the picture names they had just learnt. Pictures were presented visually again in a random order and participants were asked to provide their names. Independently of whether participants produced the picture name correctly or incorrectly, on-screen feedback was provided subsequent to their response (i.e., the words “correct” or “incorrect” were displayed on the screen accordingly), and the correct picture name was presented aurally via headphones. Once the second part of the training phase was completed, participants proceeded to the test phase.

In the reading aloud task, there was no training phase. However, 16 words that had similar characteristics as the experimental words served as practice trials. A total of 72 experimental words were then presented to each participant in a different random order.

### Apparatus and procedure

Participants were tested individually in a quiet room, seated approximately 40 cm in front of a CRT monitor. Stimulus presentation and data recording were controlled by DMDX software (Forster and Forster, 2003). Verbal responses were recorded by a head-worn microphone. In the picture naming task, participants were told that they would see the same pictures that they had previously been familiarized with and that their task was to name each picture as quickly and as accurately as possible, without hesitation. The pictures appeared on a white background in the center of the screen and remained there for 2000 ms. The 72 pictures were presented to each participant in a different random order.

In the reading aloud task, participants were told that they would be shown a series of words and that their task was to read aloud each word as quickly and as accurately as possible, without hesitation. The words were presented in lowercase letters (14-point Courier New font) and appeared in black on a white background in the center of the screen for 2000 ms. Following 16 practice trials, the 72 words were presented to each participant in a different random order.

## Results

Participants' reaction times (RTs) in both the picture naming and reading aloud tasks were hand-marked using CheckVocal (Protopapas, 2007). Incorrect responses, mispronunciations, and hesitations (2.3% of the data in the picture naming task and 0.6% of the data in the reading aloud task) were treated as errors and discarded. Initial-phoneme durations and whole-word durations were measured using Praat (Boersma, 2001). Due to microphone clipping and mobile interference, 5.3% of the data in the picture naming task and 2% of the data in the reading aloud task could not be properly labeled and were therefore discarded. The hand-marking of participants' RTs and the acoustic labeling of initial-phoneme and whole-word durations were both performed by an independently trained rater who was naïve to the purposes of the experiment. The picture naming and reading aloud data were initially combined in a single analysis.

### Reaction times

To control for temporal dependencies between successive trials, the RT of the previous trial was taken into account in the analyses, so trials whose previous trial corresponded to an error and participants' first trial in each task (2.6% of all data) were excluded. The analyses were performed using linear mixed effects models (Baayen, 2008; Baayen et al., 2008) and the languageR (Baayen, 2008), lme4 1.0-5 (Bates et al., 2013), MASS (Venables and Ripley, 2002), and lmerTest (Kuznetsova et al., 2013) packages implemented in R (R Core Team, 2014, version 3.1.2).

The Box-Cox procedure indicated that inverse RT (1/RT) was the optimal transformation to meet the precondition of normality. We then multiplied 1/RT by −1000 (−1000/RT) to maintain the direction of effects, so that a larger inverse RT meant a slower response. In our model, inverse RT was the dependent variable. The fixed effects included the interaction between frequency type (HF vs. LF) and task type (picture naming vs. reading aloud), AoA, the RT of the previous trial, and trial order. The frequency type factor and the task type factor were both deviation-contrast coded (−0.5, 0.5) to reflect the factorial design. Intercepts for subjects and items were included as random effects.

The results (obtained from 3990 observations) indicated a significant main effect of frequency: LF items were named slower than HF items (*t* = 6.80, *p* < 0.001). There was also a significant main effect of task: RTs were significantly faster in reading aloud compared to picture naming (*t* = −16.40, *p* < 0.001). The effect of AoA was also significant (*t* = 3.89, *p* < 0.001), and so were the effects of the RT of the previous trial (*t* = 3.49, *p* < 0.001) and trial order (*t* = 8.57, *p* < 0.001). Importantly, frequency type interacted with task type (*t* = −4.49, *p* < 0.001), as the size of the frequency effect was significantly larger in picture naming compared to reading aloud (55 vs. 12 ms).

The picture naming and reading aloud tasks were then analyzed separately. In the analysis of the picture naming data, the fixed effects included frequency type (HF vs. LF), AoA, the RT of the previous trial, and trial order. Intercepts for subjects and items, and random slopes for the effect of frequency (for subjects) were included as random effects^3^. The results from this analysis (obtained from 1929 observations) showed a significant frequency effect, with LF items named slower than HF items (*t* = 5.03, *p* < 0.001), a significant effect of AoA (*t* = 4.57, *p* < 0.001), and a significant effect of trial order (*t* = 5.86, *p* < 0.001). The effect of the RT of the previous trial was not significant (*t* = 1.40, *p* > 0.05). In the analysis of the reading aloud data, frequency type (HF vs. LF), AoA, the RT of the previous trial, and trial order were included as fixed effects, and intercepts for subjects and items were included as random effects. The results (obtained from 2061 observations) showed a significant frequency effect with LF items read aloud slower than HF items (*t* = 3.51, *p* < 0.001), a significant effect of trial order (*t* = 6.47, *p* < 0.001), and a significant effect of the RT of the previous trial (*t* = 7.72, *p* < 0.001). The effect of AoA was not significant (*t* = 1.02, *p* > 0.05). The mean RTs for HF and LF items in the picture naming and reading aloud tasks are shown in Table 2.

**Table 2:** **Mean reaction times (RTs), initial-phoneme durations (IP durations), whole-word durations (WW durations), and Frequency effect (in milliseconds) in the picture naming and reading aloud tasks**.

	**RTs**			**IP durations**			**WW durations**
	**HF**	**LF**	**Freq effect**	**HF**	**LF**	**Freq effect**	
Picture naming	664	719	55	58	60	2	358
Reading aloud	467	479	12	65	66	1	406

### Initial-phoneme durations

The rater labeled the acoustic boundaries of the initial phoneme in each word via visual inspection of the waveform and spectrogram using the criteria established in the ANDOSL database (Croot et al., 1992). The analyses of the initial-phoneme durations were performed using the same version of R and the same R packages as those used in the analyses of the RT data. The Box-Cox procedure indicated that the logarithmic transformation was the best transformation for initial-phoneme durations to approach a normal distribution. Therefore, the logarithmic transformation of initial-phoneme duration was the dependent variable, while the fixed effects included the interaction between frequency type (HF vs. LF) and task type (picture naming vs. reading aloud). The frequency type factor and the task type factor were both deviation-contrast coded (−0.5, 0.5) to reflect the factorial design. Intercepts for subjects and items were included as random effects.

The results (obtained from 4098 observations) showed a frequency effect, with LF items yielding longer initial-phoneme durations than HF items. However, this effect only approached significance (*t* = 1.83, *p* = 0.07). The main effect of task was significant: initial-phoneme durations were significantly longer in reading aloud compared to picture naming (*t* = 2.1, *p* < 0.05). Importantly, frequency type did not interact with task type (*t* = −1.04, *p* > 0.05). As in the RT analyses, the initial-phoneme durations in the picture naming and reading aloud tasks were subsequently analyzed separately. The analyses of the picture naming data (based on 1996 observations) showed a significant frequency effect (*t* = 2.0, *p* < 0.05), with LF items yielding significantly longer initial-phoneme durations than HF items. However, the analyses of the reading aloud task (based on 2102 observations) failed to show a significant frequency effect (*t* = 0.57, *p* > 0.05). The mean initial-phoneme durations for HF and LF items in the picture naming and reading aloud tasks are shown in Table 2.

### Whole-word durations

The same rater labeled the two acoustic boundaries that defined word duration. These were placed at the onset of acoustic energy, which was similarly denoted in all speech sounds by an increase in amplitude on the waveform, and at the offset of acoustic energy. When the last sound of the word was a stop, the second acoustic boundary that marked the end of the word was placed at the end point of the stop closure. Frequency effects on whole-word durations could not be examined given that the paired items in the HF and LF lists contained different codas. Therefore, in this analysis, we examined task effects (picture naming vs. reading aloud) on whole-word duration.

The analysis was performed using the same version of R and the same R packages as those used in the analyses of the RT and initial-phoneme duration data. The Box-Cox procedure indicated that the logarithmic transformation was the best transformation for the whole-word duration data. As such, the dependent variable in this analysis was the logarithmic transformation of whole-word duration, while task type (picture naming vs. reading aloud) was included as a fixed effect and intercepts for subjects and items were the random effects. The results (obtained from 4098 observations) showed a significant effect of task: whole-word durations were significantly longer in reading aloud compared to picture naming (*t* = 3.42, *p* < 0.01). The mean whole-word durations for all items in the picture naming and reading aloud tasks are shown in Table 2.

## General discussion

Uttering a verbal response involves the combination of cognitive and articulatory processes; however, such processes have been traditionally investigated separately, perhaps due to the widely-held assumption that the relationship between cognitive and articulatory levels of processing is staged, so that articulation can only begin once a phonological code has been generated (Levelt et al., 1999; Coltheart et al., 2001). A number of studies have provided evidence that challenges this assumption. Such evidence comes from speech errors, which contain articulatory characteristics of unselected sounds; and from effects of lexical frequency, phonological neighborhood density, syntactic predictability, and semantic congruency on the acoustic realization of verbal responses. Yet the evidence in this domain is not entirely consistent.

In the present study, we investigated effects of lexical frequency on articulation using the same stimuli in a picture naming and a reading aloud task. We reasoned that if lexical frequency affects durational aspects of verbal responses (e.g., initial-phoneme duration), we can conclude that cognitive processing continues to occur after the initiation of articulation. Such an observation would support the view that information from cognitive to articulatory levels of processing flows in a cascaded rather than a staged manner. In addition, we hypothesized that in a conceptually driven task such as picture naming, lexical frequency effects on articulation would be more prominent than in reading aloud. This is because semantic activation of the target stimulus is required in picture naming, and so its associated lexical variables (e.g., word frequency) are likely to cascade down to articulation (on the assumption that there is “leakage” of lexical activation from cognitive to articulatory levels of processing). However, reading aloud can be performed, in principle, on the basis of sublexical information, and so lexical variables associated with the printed word (e.g., its frequency) are less likely to trickle down to articulatory levels of processing.

Even though the analyses of RTs were not the focus of the present research, it is worth noting that the results were as expected. In particular, we observed a robust frequency effect, so that LF items were named slower than HF items. This was the case for both picture naming and reading aloud. Interestingly, the size of the frequency effect was significantly bigger in picture naming compared to reading aloud (55 vs. 12 ms). This result is consistent with the hypothesis that in conceptually driven tasks, where there is necessarily semantic activation of the target item, lexical variables associated with the target (e.g., word frequency) may have a robust effect on verbal responses (be that an effect on response latencies or durations)^4^. We also observed that response latencies were overall slower in picture naming than in reading aloud, a finding that was first observed over a century ago (Cattell, 1885).

The analyses of initial-phoneme durations, which were the focus of the present research, were overall consistent with the findings from previous studies that investigated effects of lexical frequency on acoustic durations (e.g., Pluymaekers et al., 2005a; Gahl, 2008; etc.). In particular, LF items yielded longer initial-phoneme durations than HF items, yet the size of this effect was very small and missed significance. Separate analyses of the picture naming and reading aloud data revealed a significant frequency effect on initial-phoneme durations for picture naming but not for reading aloud. Even though this finding is consistent with our hypothesis, namely that lexical frequency effects on articulation should be more prominent in picture naming than in reading aloud, the small size of this effect (2 ms) in combination with the absence of a significant interaction between frequency and task does not allow us to firmly conclude that lexical frequency trickles down to affect articulatory levels of processing in speech production.

Furthermore, we observed that both initial-phoneme and whole-word durations were significantly longer in reading aloud than in picture naming. This finding is consistent with the findings of Hennessey and Kirsner (1999) who reported that response durations of the same words were longer in reading aloud than in picture naming (for LF items only). To explain their findings, the authors postulated that reading aloud is initiated on the basis of partial information from the printed word. Because of this early start, the computation of phonology of the rest of the word needs be carried out during response execution, thus resulting in longer response durations in this task compared to picture naming. This account could explain our data. If response execution in reading aloud is stretched out to compensate for an early start, we may observe that in our reading aloud data, faster RTs are associated with longer initial-phoneme and whole-word durations. As we expected, the nature of the relationship between RTs and initial-phoneme durations, and RTs and whole-word durations in the reading aloud task was negative, however the correlation was weak in both cases (*r* = −0.27, *p* < 0.001, and *r* = −0.06, *p* < 0.01, respectively).

To conclude, the present study investigated effects of lexical frequency on articulation using the same stimuli in a picture naming and a reading aloud task. In agreement with previous studies, we obtained longer initial-phoneme durations for LF items than for HF items. However, the observed frequency effect reached significance only in the picture naming task. Our data suggest that high levels of cognitive processing influence, to some extent, low levels of articulatory processing. Yet, given the small size of the effect, we are reluctant to draw firm conclusions about whether the nature of the relationship between cognitive and articulatory levels of processing in speech production is cascaded or staged.

## Author note

Ethics approval for this research was obtained from the department of Psychology at Royal Holloway, University of London (2012/086). The authors would like to thank Eva Liu for hand-marking participants' response latencies and labeling the acoustic boundaries of participants' response durations. This research was supported by a British Academy Postdoctoral Fellowship to the first author. The second author was supported by a research grant from the Economic and Social Research Council (ES/L002264/1).

### Conflict of interest statement

The authors declare that the research was conducted in the absence of any commercial or financial relationships that could be construed as a potential conflict of interest.

## Footnotes

^1^Frequency values were obtained from SUBTLEX-UK (Van Heuven et al., 2014). These values are expressed on a Zipf scale. Values 1–3 correspond to LF words and values 4–7 correspond to HF words. We also obtained frequency values from SUBTLEX-US (Brysbaert and New, 2009). These values are expressed per million. According to the frequency values obtained from SUBTLEX-US, the items in the HF list were also significantly higher in frequency than the items in the LF list [*t*_(35)_ = 8.05, *p* < 0.001].

^2^It is worth noting that due to an oversight, the American names of two of the objects were used in the study (*robe* instead of *gown* and *pants* instead of *trousers*). However, given that a training phase preceded the test phase, participants were already familiarized with the names of these two objects before carrying out the task.

^3^The more complex model that included random slopes for the effect of frequency for subjects had a significantly better fit [χ^2^(2) = 5.99, Pr(>Chisq) = 0.05], hence this model was preferred over the simpler model.

^4^Taikh et al. (2015) recently published semantic decision times from a study in which participants saw a series of pictures (or a series of words) one at a time on the screen, and had to decide whether each represents something living or nonliving. Thirty-two of our stimuli overlapped with the items used in the Taikh et al. (2015) study. If picture naming involves semantic activation of the target stimuli, picture naming RTs for these 32 items in our study should correlate with semantic decision times for the same pictures in the Taikh et al. study. However, reading aloud RTs for these 32 items in our study may not correlate with semantic decision times for the same words in the Taikh et al. study. This was the case; the correlation between our picture naming RTs and their semantic decision times for the 32 pictures was significant (*r* = 0.36, *p* < 0.05), whereas the correlation between our reading aloud RTs and their semantic decision times for the 32 words was not (*r* = 0.16, *p* > 0.05). We thank Marc Brysbaert for pointing us to the Taikh et al. (2015) article and for suggesting this analysis.
