# Supplementary material for: Lexical frequency effects on articulation: a comparison of picture naming and reading aloud
Source: Front Psychol. 2015 Oct 15;6:1571. doi: 10.3389/fpsyg.2015.01571 (PMC4606046; doi:10.3389/fpsyg.2015.01571)
Supplement: Supplementary file 1 [file DataSheet1.DOCX]

Appendix. Paired HF and LF items used in the picture naming and reading aloud tasks.

| HF | LF |
| --- | --- |
| bag | bat |
| beach | bean |
| bed | bell |
| bin | bib |
| boat | bone |
| brain | braid |
| bus | bug |
| cat | cap |
| clock | clog |
| coat | cone |
| corn | cork |
| crash | crab |
| dog | doll |
| fish | fig |
| hat | ham |
| leaf | leek |
| map | mat |
| moon | moose |
| mouth | mouse |
| neck | net |
| pig | pill |
| pipe | pine |
| plane | plate |
| plug | plum |
| rain | rake |
| ring | rib |
| road | robe |
| rock | rod |
| roof | root |
| sheep | sheath |
| snake | snail |
| stone | stove |
| train | trace |
| trap | tram |
| wheel | wheat |
| witch | wig |
